# Clonal outbreak of an extensively drug-resistant NDM-1 producing *Pseudomonas aeruginosa* in a local hospital in the Czech Republic

**DOI:** 10.1128/spectrum.02581-25

**Published:** 2025-12-03

**Authors:** Katerina Chudejova, Tsolaire Sourenian, Marc Finianos, Anna Sramkova, Costas C. Papagiannitsis, Jaroslav Hrabak, Ibrahim Bitar

**Affiliations:** 1 Department of Microbiology, Faculty of Medicine, University Hospital in Pilsen, Charles University, Pilsen, Czech Republic; 2 Department of Microbiology, University Hospital of Larissa, Larissa, Greece; Universita degli Studi Roma Tre, Rome, Italy

**Keywords:** *Pseudomonas aeruginosa*, ST773, *bla*_NDM-1_, outbreak, XDR

## Abstract

A clonal outbreak of 18 ST773 NDM-1 producing *Pseudomonas aeruginosa* strains has been detected for the first time in the Czech Republic. The strains were extremely drug-resistant (XDR) and resistant to serum killing. SNP-based phylogeny and CRISPR assay typing showed minimal genomic variations among the isolates. The results suggest a high-risk, persistent, virulent clone causing the hospital outbreak, with the possibility of a nationwide outbreak.

**IMPORTANCE:**

Our research on the novel detection of the NDM-1 gene in carbapenem-resistant *Pseudomonas aeruginosa* ST773 in the Czech Republic is of great significance for public health and infection control. Until now, the emergence of this gene in *P. aeruginosa* strains was uncommon in this region, as carbapenem resistance was primarily associated with IMP and VIM types of MBLs. This nosocomial outbreak was triggered by an index case patient repatriated from areas with reported NDM-1 producing *P. aeruginosa*, illustrating how international travel contributes to the spread of such resistant pathogens. The results obtained in this study show that it is necessary to focus on tracing the source of infections to control and prevent nosocomial infections, helping to protect public health in the Czech Republic.

## OBSERVATION

The worldwide spread of carbapenem-resistant *Pseudomonas aeruginosa*, which frequently belongs to international epidemic high-risk clones widely disseminated in hospital settings, has become an emerging challenge in the last years (1, 2). The most important mechanisms of resistance to carbapenems in *P. aeruginosa* are the production of carbapenemases (3, 4). The most frequently described carbapenemases in *P. aeruginosa* are metallo-beta-lactamases, such as IMP and VIM, and less often NDM (4). In the Czech Republic in 2015, a comprehensive study on 194 carbapenem-resistant *P. aeruginosa* from 43 health-care facilities confirmed the highest prevalence of IMP metallo-beta-lactamase, followed by VIM type and GES type (3), but no NDM type has been detected. Up to this time, several cases of *P. aeruginosa* ST773 NDM-1 (PA-NDM-1) spread were described worldwide. The first case was reported by Kocsis et al. in Hungary (5), followed by reports from the USA (6, 7), India (8), Nepal (8), the Netherlands (9), and most recently from Spain (10). In this study, we describe the first case of an imported extremely drug-resistant (XDR) *P. aeruginosa* isolate of the high-risk clone ST773 harboring the *bla*_NDM-1_ gene and causing a local outbreak in a hemato-oncology department of a Czech university hospital.

The first case of a PA-NDM-1 isolate was reported in May 2022 from a rectal swab of a male patient suffering from acute myeloblastic leukemia. He was repatriated from Ukraine and admitted to the hemato-oncology department of the University Hospital in Pilsen. Prior to the admission, he underwent a bone marrow transplantation in Turkey in the spring of 2021 and was subsequently taken back into care in Ukraine. Since the admission in May 2022, a surge in the number of NDM-producing *P. aeruginosa* isolates has been recorded (up until January 2023). Another 17 patients with PA-NDM-1 were detected in the same hemato-oncology department. Due to the severity of the primary diseases and the limited options of antibiotic therapy, five patients who developed bacterial sepsis died. During the same period, two more cases were detected in other Czech hospitals. The first one was reported from a patient in the University Hospital in Hradec Kralove. The patient, who has been living long-term in Tunisia, was hospitalized there due to a car accident for 10 days and then transferred to Hradec Kralove. The second case was reported from a patient repatriated from Ukraine in a surgery ambulance of Hospital Ceske Budejovice (Table S1).

Species identification was done using MALDI-TOF MS. Antibiotic susceptibility testing was performed using broth microdilution assay according to the EUCAST (2025) guidelines, and results were interpreted according to its 2025 breakpoints criteria (http://www.eucast.org/). Carbapenemase production was confirmed using the MALDI-TOF MS meropenem hydrolysis assay (11), phenotypic detection of carbapenemase type was done using the double-disc synergy test, and the PCR amplification of genes encoding carbapenemases was performed as described previously (12). All isolates were *P. aeruginosa* harboring *bla*_NDM_. All isolates showed an XDR profile showing resistance against most of the tested antibiotics, such as ampicillin-sulbactam, piperacillin-tazobactam, ceftazidime, meropenem, and ciprofloxacin, yet were susceptible against aztreonam, colistin, and cefiderocol (Table S2).

Genomic DNAs from all isolates were sequenced using a short-read sequencing platform on NovaSeq 600 (Illumina, Inc., USA). All reads, assembled using SPAdes v3.14.0 (13), were analyzed using public databases in the Center for Genomic Epidemiology (https://genomicepidemiology.org/) (14) (Table 1). All isolates belonged to sequence type ST773 and harbored genes coding for resistance against ciprofloxacin (*qnrVC1*), aminoglycoside (*rmtB*, *aph(3’)-llb*), sulfamethoxazole (*sul1*), fosfomycin (*fosA*), tetracycline (*tet(G*)), amphenicol (*catB7*), and beta-lactams, including carbapenems (intrinsic *bla*_PAO_ and most importantly *bla*_NDM-1_). Furthermore, all isolates harbored genes coding for quorum sensing (*lasI*, *lasR*, *rhlI*, *rhlR*, *hdtS*) and adherence (such as *pilB*, *pilD-U*, *pilZ,* and *fimV*). Moreover, isolates harbored genes coding for anti-phagocytosis (*alg44*, *alg8*, *algA-X*, *mucA-E,* and *mucP*). These isolates were further tested through analyzing their *in vitro* virulence by testing the survival of bacteria in pooled human serum (NHS) or heat-inactivated normal human serum (HI-NHS) as described elsewhere (15). We found that all *P. aeruginosa* isolates, except isolate CZ75789, which is not part of the outbreak, exhibited resistance to killing by complement (Fig. S1).

**TABLE 1:** WGS analysis including sequence type, resistance, and selected groups of virulence genes in all the sequenced *P. aeruginosa* isolates carrying the *bla*_NDM-1_ gene

Isolate	Sequence type	Resistance genes	Quorum sensing	Immune modulation	Adherence	Toxins
CZI1002861	773	*bla*_NDM-1_*;* *bla*_OXA-395_*;* *bla*_PDC-16_*;* *aadA10;* *aph(3')-IIb;* *rmtB;* *qnrVC1;* *sul1;* *fosA;* *catB7;* *tet(G*)	*lasI;* *lasR;* *rhlI;* *rhlR* *hdtS*	*alg44; alg8;* *algA-X;* *mucA-E;* *mucP*	*fleN; fleQ; fleR; fleS; flgA-N; flhA; flhB; flhF; fliA-T; pilB, pilD-U, pilY1, pilZ, motA-D; motY; fimV, chpA-E*	*exoU; exoT; exoY; toxA*
CZH83072	773					
CZI1013241/4	773					
CZI1013428/4	773					
CZH86815	773					
CZI1014516/3	773					
CZI1015194/4	773					
CZI1016148	773					
CZI1017058	773					
CZI1017991	773					
CZH89882	773					
CZI1019629	773					
CZI1019706	773					
CZ75475	773					
CZ75789	773					
CZI1022513/4	773					
CZI1023419	773					
CZI1025008	773					
CZI1025809/5	773					
CZI1026033	773					

DNA isolated from the index case strain (CZI1002861) and from another strain (CZI1023419), collected at the end of the collection period (after 6 months), were sequenced on PacBio Sequel I (Pacific Biosciences, USA) to produce complete circular genomes and for the purpose of deep genomic comparison for possible detection of microevolution. Long-read WGS data showed that the *bla*_NDM-1_ was inserted into the *P. aeruginosa* chromosome. The *bla*_NDM-1_ gene was located within the integrative and conjugative element (ICE) ICE*6600-like*, as previously described in the *P. aeruginosa* strain P-600 (GenBank accession no. CP053917) isolated from South Korea.

Furthermore, the clonality of the isolates of this outbreak was determined by CRISPR array typing as previously described (16). All isolates had the CRISPR/Cas I-E type with an identical CRISPR array (100%).

**Fig 1:**
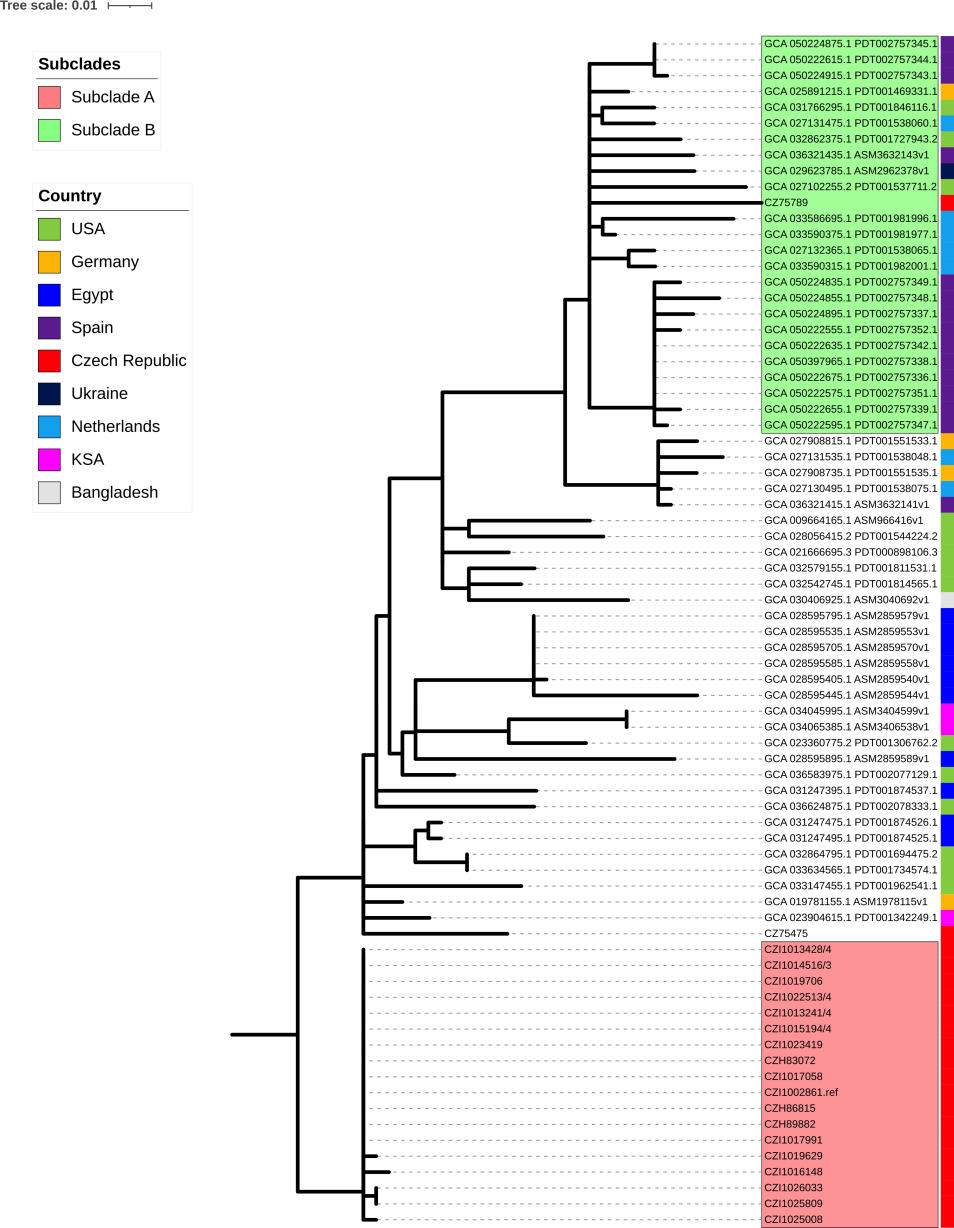
SNP-based phylogeny for all *P. aeruginosa* ST773 genomes in the NCBI database. Clades A and B are highlighted in red and green, respectively. The outermost colored bar to the right corresponds to the isolates’ reporting country.

Finally, SNP-based phylogeny for all *P. aeruginosa* ST773 in the NCBI database (along with our isolates) was done as described previously (16) (index case was used as a reference). The phylogeny grouped the outbreak isolates into one clade (clade A) (Fig. 1), while the isolate from Hradec Kralove belonged to another clade (clade B) of ST773 isolates. The ST773 isolate from Budejovice wasn’t grouped in any clade. The SNPs between the genomes in comparison with the genome of the index case ranged from 0 to 11 among the isolates of the outbreak. While it was higher with the ones from Hradec Kralove and Budejovice (56 and 333, respectively) (Table S3). These results strongly suggest that the outbreak was caused by one strain.

The hospitals implemented immediate infection control measures to control the outbreak through isolation/quarantine of colonized patients separately or in groups, depending on the initial diagnosis of the patient. Moreover, they increased cleaning/sanitary intervention methods, staff and patients education, and continuous consultation with local epidemiologists. These measures managed to limit the dissemination of this clone.

In conclusion, in the current study, we describe the emergence of an outbreak due to the spread of ST773 *P. aeruginosa* isolates producing NDM-1 carbapenemase in Pilsen hospital. The outbreak took place due to the repatriation of a patient previously hospitalized in Ukraine. Additionally, two sporadic cases of ST773 *P. aeruginosa* isolates producing NDM-1 were identified in different hospitals. These findings, which are in agreement with the increased reports in literature reporting the isolation of ST773 *P. aeruginosa* isolates producing NDM-1 in several countries, highlight the development of ST773 as a high-risk clone.

## Data Availability

All genomes have been deposited in the GenBank under the Bio Project accession number PRJNA1048319.

## SUPPLEMENTAL MATERIAL

Figure S1 spectrum.02581-25-s0001.docx Survival of *P. aeruginosa* isolates in 80% NHS and heat-inactivated NHS. The presented data is analyzed according to log (CFU 3 hrs/CFU 0 hours).

Table S1 spectrum.02581-25-s0002.docx The descriptive data of the ST773 NDM-1 producing *P. aeruginosa* isolates from the Czech Republic. This data includes isolation date, specimen type, hospital, the ward of the isolation, patient diagnosis, and the travel history.

Table S2 spectrum.02581-25-s0003.docx Antibiotic susceptibility profiles for the ST773 NDM-1 producing *P. aeruginosa* isolates from the Czech Republic.

Table S3 spectrum.02581-25-s0004.xls SNP analysis and related data of the genomes of this study when compared to the genome of the index case.

ASM does not own the copyrights to Supplemental Material that may be linked to, or accessed through, an article. The authors have granted ASM a non-exclusive, world-wide license to publish the Supplemental Material files. Please contact the corresponding author directly for reuse.
