# Supplementary material for: Clonal outbreak of an extensively drug-resistant NDM-1 producing Pseudomonas aeruginosa in a local hospital in the Czech Republic
Source: Microbiol Spectr. 2025 Dec 3;14(1):e02581-25. doi: 10.1128/spectrum.02581-25 (PMC12772245; doi:10.1128/spectrum.02581-25)
Supplement: Figure S1 — Survival of P. aeruginosa isolates in 80% NHS and heat-inactivated NHS. The presented data is analyzed according to log (CFU 3 hrs/CFU 0 hours). [file spectrum.02581-25-s0001.docx]

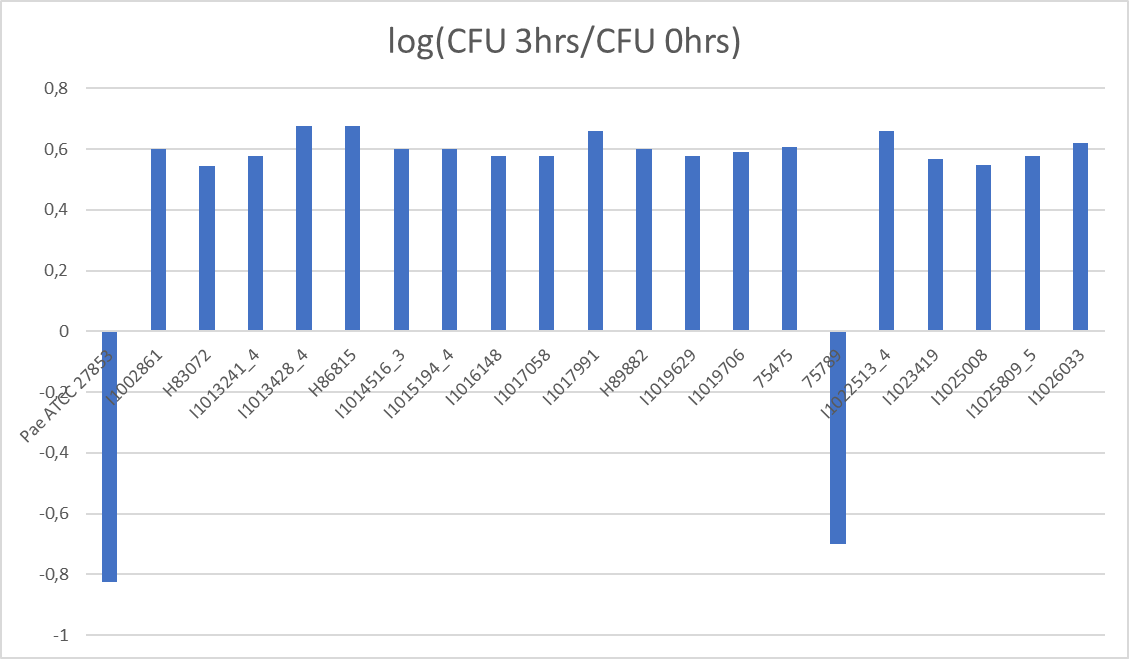


Figure S1: Survival of *P. aeruginosa* isolates in 80% NHS and heat inactivated NHS. The presented data is analyzed according to log (CFU 3hrs/CFU 0hours).
