# Supplementary material for: Clonal outbreak of an extensively drug-resistant NDM-1 producing Pseudomonas aeruginosa in a local hospital in the Czech Republic
Source: Microbiol Spectr. 2025 Dec 3;14(1):e02581-25. doi: 10.1128/spectrum.02581-25 (PMC12772245; doi:10.1128/spectrum.02581-25)
Supplement: Table S1 — The descriptive data of the ST773 NDM-1 producing P. aeruginosa isolates from the Czech Republic. This data includes isolation date, specimen type, hospital, the ward of the isolation, patient diagnosis, and the travel history. [file spectrum.02581-25-s0002.docx]

| **Isolate** | **Isolation date** | **Specimen** | **Hospital** | **Ward** | **Diagnosis** | **Travel/Medical history** | **Live status in connection to PSAE infection** | **Carbapenem treatment** | **Hospitalization period**  **(Month)** |
| --- | --- | --- | --- | --- | --- | --- | --- | --- | --- |
| CZI1002861 | 23.5.2022 | rectal swab | University Hospital Pilsen | Hematooncology | Acute myeloblastic leukemia | Ukraine/Turkey | alive | meropenem | 5 |
| CZH83072 | 2.7.2022 | hemoculture | University Hospital Pilsen | Hematooncology | Acute myeloblastic leukemia | none | dead | no | 2 |
| CZI1013241/4 | 11.8.2022 | urine | University Hospital Pilsen | Hematooncology | Mediastinal large B-cell lymphoma | none | alive | no | 4 |
| CZI1013428/4 | 13.8.2022 | rectal swab | University Hospital Pilsen | Hematooncology ICU | Other transplanted organ and tissue status | none | alive | meropenem | 4 |
| CZH86815 | 15.8.2022 | hemoculture | University Hospital Pilsen | Hematooncology ICU | Other myelodysplastic syndromes | none | dead | meropenem | 3 |
| CZI1014516/3 | 23.8.2022 | rectal swab | University Hospital Pilsen | Hematooncology ICU | Acute promyelocytic leukemia | none | alive | no | 1 |
| CZI1015194/4 | 29.8.2022 | rectal swab | University Hospital Pilsen | Hematooncology | Mixed cellularity Hodgkin lymphoma | none | alive | no | 1 |
| CZI1016148 | 6.9.2022 | rectal swab | University Hospital Pilsen | Hematooncology ICU | Acute myeloblastic leukemia | none | alive | meropenem | 7 |
| CZI1017058 | 13.9.2022 | hemoculture | University Hospital Pilsen | Hematooncology ICU | Acute myeloblastic leukemia | none | dead | no | 1 |
| CZI1017991 | 20.9.2022 | rectal swab | University Hospital Pilsen | Hematooncology ICU | Acute myeloblastic leukemia | none | alive | meropenem | 4 |
| CZH89882 | 23.9.2022 | hemoculture | University Hospital Pilsen | Hematooncology | Other myelodysplastic syndromes | none | dead | no | 3 |
| CZI1019629 | 3.10.2022 | Permanent urine catheter | University Hospital Pilsen | General ICU | Sepsis | none | alive | meropenem | 1 |
| CZI1019706 | 3.10.2022 | rectal swab | University Hospital Pilsen | Hematooncology | Aplastic anemia, unspecified | none | alive | no | 2 |
| CZ75475 | 7.10.2022 | rectal swab | University Hospital Hradec Kralove | Neurosurgery ICU | polytrauma, craniotrauma after car accident | Tunisia | alive | meropenem | 3 |
| CZ75789 | 19.10.2022 | wound swab | Ceske Budejovice Hospital | surgery ambulance | Encounter for observation for other suspected diseases and conditions ruled out | Ukraine | alive | no | 2 |
| CZI1022513/4 | 25.10.2022 | hemoculture | University Hospital Pilsen | Hematooncology | Acute myeloblastic leukemia | Ukraine | dead | no | 1 |
| CZI1023419 | 1.11.2022 | urine | University Hospital Pilsen | Hematooncology | Acute promyelocytic leukemia | none | alive | meropenem | 2 |
| CZI1025008 | 12.11.2022 | rectal swab | University Hospital Pilsen | Hematooncology | Mantle cell lymphoma | none | alive | no | 2 |
| CZI1025809/5 | 18.11.2022 | rectal swab | University Hospital Pilsen | Hematooncology | Diffuse large B-cell lymphoma | none | alive | meropenem | 8 |
| CZI1026033 | 21.11.2022 | urine | University Hospital Pilsen | Hematooncology | Acute myeloblastic leukemia | none | alive | meropenem | 3 |

**Table S1:** The descriptive data of the ST773 NDM-1 producing *P. aeruginosa* isolates from the Czech Republic. This data includes isolation date, specimen type, hospital, the ward of the isolation, patient diagnosis and the travel history.
