# Supplementary material for: Clonal outbreak of an extensively drug-resistant NDM-1 producing Pseudomonas aeruginosa in a local hospital in the Czech Republic
Source: Microbiol Spectr. 2025 Dec 3;14(1):e02581-25. doi: 10.1128/spectrum.02581-25 (PMC12772245; doi:10.1128/spectrum.02581-25)
Supplement: Table S2 — Antibiotic susceptibility profiles for the ST773 NDM-1 producing P. aeruginosa isolates from the Czech Republic. [file spectrum.02581-25-s0003.docx]

**Table S2:** Antibiotic susceptibility profiles for the ST773 NDM-1 producing *P. aeruginosa* isolates from the Czech Republic.

| **Isolate** | **MIC (mg/L)** | | | | | | | | | | | | **DDT (mm)** |
| --- | --- | --- | --- | --- | --- | --- | --- | --- | --- | --- | --- | --- | --- |
|  | **Sam** | **Pip** | **Tzp** | **Caz** | **Atm** | **Mem** | **Gm** | **Amk** | **Col** | **Cip** | **Tgc** | **Sxt** | **Fdc** |
| CZI1002861 | >128 | 64 | 64 | >16 | 1 | >16 | >32 | >64 | **1** | >8 | 2 | >4 | **26** |
| CZH83072 | >128 | 64 | 64 | >16 | 2 | >16 | >32 | >64 | **1** | >8 | 4 | >4 | **24** |
| CZI1013241/4 | >128 | 128 | 128 | >16 | 2 | >16 | >32 | >64 | **1** | >8 | >8 | >4 | **24** |
| CZI1013428/4 | >128 | 128 | 128 | >16 | 2 | >16 | >32 | >64 | **1** | >8 | 4 | >4 | **24** |
| CZH86815 | >128 | 64 | 64 | >16 | 2 | >16 | >32 | >64 | **1** | >8 | 8 | >4 | **25** |
| CZI1014516/3 | >128 | 128 | 64 | >16 | 1 | >16 | >32 | >64 | **1** | >8 | 8 | >4 | **23** |
| CZI1015194/4 | >128 | 64 | 64 | >16 | 1 | >16 | >32 | >64 | **1** | >8 | 4 | >4 | **26** |
| CZI1016148 | >128 | 64 | 128 | >16 | 1 | >16 | >32 | >64 | **1** | >8 | 4 | >4 | **25** |
| CZI1017058 | >128 | 128 | 64 | >16 | 0,5 | >16 | >32 | >64 | **2** | >8 | 8 | >4 | **24** |
| CZI1017991 | >128 | 64 | 64 | >16 | 2 | >16 | >32 | >64 | **1** | >8 | 4 | >4 | **24** |
| CZH89882 | >128 | 128 | 128 | >16 | 1 | >16 | >32 | >64 | **1** | >8 | 4 | >4 | **25** |
| CZI1019629 | >128 | 32 | 32 | >16 | 1 | >16 | >32 | >64 | **2** | >8 | 8 | >4 | **24** |
| CZI1019706 | >128 | 32 | 64 | >16 | 1 | >16 | >32 | >64 | **1** | >8 | 2 | >4 | **25** |
| CZ75475 | >128 | 128 | 128 | >16 | 1 | >16 | >32 | >64 | **2** | >8 | >8 | >4 | **24** |
| CZ75789 | >128 | 128 | 128 | >16 | 1 | >16 | >32 | >64 | **1** | >8 | >8 | >4 | **24** |
| CZI1022513/4 | >128 | 64 | 64 | >16 | 1 | >16 | >32 | >64 | **1** | >8 | 8 | >4 | **25** |
| CZI1023419 | >128 | 128 | 128 | >16 | 2 | >16 | >32 | >64 | **1** | >8 | 8 | >4 | **25** |
| CZI1025008 | >128 | 64 | 64 | >16 | 1 | >16 | >32 | >64 | **1** | >8 | 4 | >4 | **25** |
| CZI1025809/5 | >128 | 128 | 128 | >16 | 2 | >16 | >32 | >64 | **2** | >8 | 8 | >4 | **25** |
| CZI1026033 | >128 | 128 | 128 | >16 | 2 | >16 | >32 | >64 | **2** | >8 | 8 | >4 | **25** |

MIC, minimum inhibitory concentration; Sam, ampicillin-sulbactam; Pip, piperacillin; Tzp, piperacillin-tazobactam; Caz, ceftazidime; Atm, aztreonam; Mem, meropenem; Gm, gentamicin; Amk, amikacin; Col, colistin; Cip, ciprofloxacin; Tgc, tigecycline; Sxt, trimethoprim-sulfamethoxazole; DDT, disk diffusion test; Fdc, cefiderocol.

Bold and underlined values correspond to susceptible phenotype.
